# Guttiferone K Exerts the Anti-inflammatory Effect on Mycobacterium Tuberculosis- (H37Ra-) Infected Macrophages by Targeting the TLR/IRAK-1 Mediated Akt and NF-*κ*B Pathway

**DOI:** 10.1155/2020/8528901

**Published:** 2020-10-10

**Authors:** Qingwen Zhang, Jinxia Sun, Yan Fu, Weigang He, Yinhong Li, Hongsheng Tan, Hongxi Xu, Xin Jiang

**Affiliations:** ^1^Center for Traditional Chinese Medicine and Immunology Research, School of Basic Medical Sciences, Shanghai University of Traditional Chinese Medicine, 201203 Shanghai, China; ^2^Shanghai Key Laboratory of Molecular Imaging, Shanghai University of Medicine & Health Sciences, 201318 Shanghai, China; ^3^Clinical Research Center, Shanghai Jiao Tong University School of Medicine, 200240 Shanghai, China; ^4^Shuguang Hospital, Shanghai University of Traditional Chinese Medicine, Shanghai 201203, China; ^5^School of Pharmacy, Shanghai University of Traditional Chinese Medicine, 201203 Shanghai, China

## Abstract

*Mycobacterium tuberculosis* (Mtb) remains a great threat to global health, killing more people than any other single infectious agent and causing uncontrollable inflammation in the host. Poorly controlled inflammatory processes can be deleterious and result in immune exhaustion. The current tuberculosis (TB) control is facing the challenge of drugs deficiency, especially in the context of increasingly multidrug resistant (MDR) TB. Under this circumstance, alternative host-directed therapy (HDT) emerges timely which can be exploited to improve the efficacy of TB treatment and disease prognosis by targeting the host. Here, we established the *in vitro* infection model of Mtb macrophages with H37Ra strain to seek effective anti-TB active agent. The present study showed that Guttiferone K, isolated from *Garcinia yunnanensis*, could significantly inhibit Mtb-induced inflammation in RAW264.7 and primary peritoneal macrophages. It was evidenced by the decreased production of inflammatory mediators, including interleukin-1*β* (IL-1*β*), tumor necrosis factor-*α* (TNF-*α*), interleukin-6 (IL-6), inducible nitric oxide synthase (iNOS), and cyclooxygenase-2 (COX-2). Further studies with immunoblotting and immunofluorescence revealed that Guttiferone K obviously inhibits the nuclear factor-kappa B (NF-*κ*B) both in RAW264.7 and primary peritoneal macrophages relying on the TLR/IRAK-1 pathway. Guttiferone K could also suppress the NLRP3 inflammasome activity and induce autophagy by inhibiting the protein kinase B (p-Akt) and mammalian target of rapamycin (mTOR) phosphorylation at Ser473 and Ser2448 in both cell lines. Thus, Guttiferone K possesses significant anti-inflammatory effect, alleviating Mtb-induced inflammation with an underlying mechanism that targeting on the TLR/IRAK-1 pathway and inhibiting the downstream NF-*κ*B and Akt/mTOR signaling pathways. Together, Guttiferone K can be an anti-inflammatory agent candidate for the design of new adjunct HDT drugs fighting against tuberculosis.

## 1. Introduction

Tuberculosis (TB) is the leading infectious disease in the world, which is caused by *Mycobacterium tuberculosis* (Mtb). It has killed 1.6 million people and infected another 10 million in the past year [1]. Inflammation and immunity are host responses to infection that influence the progression of the disease. The outcome of disease depends on the balance between effector and regulatory immune mechanisms. The contribution of nonresolving inflammation to pathogenesis is worth emphasizing when the host inflammatory response, rather than toxins from the pathogen, is primarily responsible for the damage to the host. Globally, TB is probably the most prevalent example. Chronic inflammation surrounding Mtb can persist for decades. When the inflammation is extensive enough, it liquefies the lung. In the past few years, an innovative and potentially high-reward approach, host-directed therapy (HDT), has been proposed, which can be used as an adjunct to antibiotic-based anti-TB treatment owing to a better understanding of TB pathogenesis and the characterization of the immunological mediators involved [2–4]. HDT is a therapeutic option that utilizes small molecules or biological agents that provide antimicrobial or beneficial effects in the following ways: (i) enhancing host immune defenses against the pathogen; (ii) interfering with the host mechanisms utilized by the pathogen to prevent its persistence or replication in the host tissue; (iii) targeting pathways that may contribute to the disease or immunopathology, such as those classically manifested by excessive inflammation; and (iv) regulating the local levels of host factors associated with pathogen responses. Growing evidence has indicated that using anti-inflammatory or immunomodulatory agents improves the outcome of TB [5].

The innate immune response provides the first line of host defense against invading pathogens. This response is triggered by the activation of pattern recognition receptors (PRRs), which represent a crucial link between pathogen detection and the induction of a proinflammatory cascade. Among the growing family of PRRs, toll-like receptors (TLRs) are the most widely studied and are known for playing a vital role in the recognition of Mtb. Among all TLRs, TLR2 and TLR4 have been widely demonstrated to be involved in the pathological processes caused by Mtb infection [6]. TLRs are generally considered to be proinflammatory, when interacted with specific mycobacterial structures, the signaling pathways are triggered, what is more, the adaptor molecule myeloid differentiation primary response protein 88 (MyD88) plays an important role in the process [7]. Subsequently, interleukin-1 receptor-associated kinases (IRAK) or other important molecules are recruited in a signaling cascade leading to the activation and translocation of nuclear transcription factor nuclear factor-*κ*B (NF-*κ*B) into the nucleus [7]. Among members of the IRAK family, IRAK1 is a key regulatory protein with pivotal functions in mediating immune cell activation, which can be phosphorylated by IRAK4 [8, 9]; whereas IRAK3 and IRAK2 are considered to be pseudokinases lacking catalytic activity, although they may still play important roles in signaling cascades [9, 10]. Mitogen-activated protein kinase (MAPK) signaling is also downstream of TLR/IRAK-1, which is one of the most important regulatory mechanisms in eukaryotic cells regulating key cellular processes, such as gene induction, cellular stress, and inflammatory responses. There are three principle members of MAPKs in mammals: p38, ERK1/2, and JNK [11]. It is well known that NF-*κ*B and MAPK signaling pathways are involved in inflammation-related diseases [12, 13]. The inflammasome pathway is associated with the coordinated release of cytokines such as IL-1*β* and IL-18, which also play a role in the pathogenesis of TB [14]. The Nod-like receptor pyrin domain containing 3 (NLRP3) inflammasome is a multimeric cytosolic complex, which is comprised of the adaptor protein apoptosis-associated speck-like protein containing a caspase recruiting domain (ASC) and a sensor protein NLRP3, together with the effector protein caspase-1. Increasing studies have indicated that Mtb infection can activate the NLRP3 inflammasome pathway, causing host tissue inflammatory immunopathology damage [15–17]. Strict control of the excessive or uncontrolled inflammatory immune response during TB may minimize mycobacterial dissemination and transmission without deleterious immunopathology [18]. Autophagy is a highly conserved biological process in eukaryotic cells which has been reported playing negative effect in the process of inflammation by inhibiting inflammasome activation and IL-1*β* release [19, 20] and protecting host from excessive inflammatory damage during Mtb infection [16, 21]. The activation of autophagy can be regulated by varieties of signals; the kinase mTOR is a major modulator of autophagy, which receives inputs from different signaling pathways and is a downstream target of the phosphatidylinositol 3 kinase (PI3K)/protein kinase B (Akt) pathway. The PI3K/Akt/mTOR pathway is downstream of TLR/IRAK-1 signaling, which has been recognized to negatively regulate the activation of autophagy [22].

*Garcinia* species are tropical evergreen trees and shrubs that are widely distributed in Southeastern Asia and used in traditional folk medicine to promote detoxification, relieve inflammation, or treat wounds [23]. Studies have shown that *Garcinia* species exert various bioactivities, such as antitumor, anti-inflammatory, antiviral, and neuroprotective effects [24–26]. Guttiferone K (GK) was isolated from *Garcinia yunnanensis*, and it has been found that Guttiferone K suppresses the cell motility and metastasis of hepatocellular carcinoma [27]. Moreover, Guttiferone K can induce autophagy and sensitize cancer cells to nutrient stress-induced cell death [28]. This study was conducted to investigate the potential immunological protective effect of Guttiferone K in macrophages infected with Mtb and manage to explain the mechanism of how it works. We expect to seek effective bioactive molecules that can be designed as adjunct HDT anti-TB drugs together with antibiotics to treat TB.

## 2. Material and Methods

### 2.1. Chemicals and Reagents

RIPA lysis buffer, BCA Protein Assay Kit, and Protein A/G agarose/sepharose beads were obtained from the Beyotime Institute of Biotechnology (Shanghai, China). The following antibodies were used: anti-NLRP3, anti-IL-1*β*, anti-ASC, anti-LC3, anti-p62, anti-Akt, anti-phosphorylated Akt (Ser473), anti-mTOR, anti-phosphorylated mTOR (Ser2448), anti-phosphorylated p38, anti-phosphorylated JNK, and anti-phosphorylated ERK1/2 were purchased from Cell Signaling Technology, Inc. (CST, Danvers, MA, USA); anti-IL-1*β* was purchased from R&D Systems (Minnesota, USA); BECN1 siRNA, Transfection Reagents, rabbit anti-caspase-1, goat anti-rabbit, donkey anti-goat, and goat anti-mouse LC3 were purchased from Santa Cruz Biotechnology, Inc.; anti-*β*-actin monoclonal antibody was from ProteinTech Group (Chicago, IL); goat anti-NLRP3 was purchased from Abcam (Cambridge, UK). Guttiferone K (GK, purity > 98%, C38H50O6, MW: 602.80) was isolated from *Garcinia yunnanensis* as previously described [29]; Dulbecco's Modified Eagle's Medium (DMEM) was obtained from HyClone Laboratories, Inc. (Logan, UT, USA). Middlebrook 7H9 and 7H10 media were obtained from Difco (Detroit, MI, USA), and oleic acid-albumin-dextrose-catalase (OADC) supplements were from BD Biosciences (BD, Sparks, MD, USA).

### 2.2. Animals

Animal experiments were conducted in strict accordance with the National Institute of Health Guide for the Care and Use of Laboratory Animals, with the approval of the Scientific Investigation Board of Shanghai University of Traditional Chinese Medicine (Shanghai, China). Female C57BL/6J mice (4-8 weeks, 20 ± 3 g) were obtained from Vital River Laboratory Animal Technology Co., Ltd. (Beijing, China). All mice were acclimated for at least 1 week before the experiments and housed in a pathogen-free facility.

### 2.3. Cell Culture

The RAW264.7 murine macrophage cell line was maintained in DMEM supplemented with 10% fetal bovine serum (FBS) in 5% CO_2_ at 37°C. Thioglycolate-elicited mouse primary peritoneal macrophages were prepared from female C57BL/6J mice as described previously [30]. After 2 hr, nonadherent cells were removed, and the adherent cells were used as primary peritoneal macrophages.

### 2.4. CCK-8 Assay for Cell Viability

RAW264.7 (1 × 10^4^ cells/well) were seeded into 96-well culture plates overnight at 37°C and atmospheric conditions of 5% CO_2_. The culture medium was then replaced with medium containing different concentrations of GK for 24, 48, and 72 hr. At the end of the culture, 10 *μ*l of the CCK-8 reagent was added to each well. After 1-2 hr of incubation at 37°C, the absorbance was determined at 450 nm using a Synergy 2 Microplate Reader (Bio-Tek, USA).

### 2.5. Bacterial Strains

Mtb H37Ra was used in the current study. The H37Ra strain was grown in Middlebrook 7H9 or 7H10 broth supplemented with 0.2% glycerol, 0.05% Tween-80, and 10% Middlebrook OADC supplement.

### 2.6. Mtb Infection

The RAW264.7 cells or primary peritoneal macrophages were seeded at a density of 1.0 × 10^6^ cells/well in a 6-well plate and grew at 37°C overnight. The cells were infected with Mtb H37Ra at a MOI (multiplicity of infection) of 10 : 1 and grew at 37°C overnight. After 4 hr of coincubation, cells were washed three times with sterile PBS to remove extracellular bacteria and cultured with DMEM containing 10% FBS in the presence of different concentrations of GK for different time.

### 2.7. Western Blot

Cells were collected and lysed in lysis buffer (Beyotime Institute of Biotechnology, China), and then, whole cell lysate was separated by SDS-PAGE and further transferred onto nitrocellulose membranes (Pall, USA). After blocking with TBST (0.5% Tween-20) containing 5% (w/v) nonfat milk, the membranes were incubated with specific primary antibodies at 4°C overnight in blocking solution; all antibodies were diluted at 1 : 1000. After washed with TBST for 3 times, the membranes were incubated with HRP-conjugated secondary antibodies at room temperature for 1 hr. Chemiluminescence was detected using an ECL-chemiluminescent kit (Thermo Scientific) with Protein Simple (USA).

### 2.8. Coimmunoprecipitation

Primary peritoneal macrophages were lysed at 4°C in ice-cold cell lysis buffer, and cell lysates were cleared by centrifugation (12,000*g*, 10 min). Before immunoprecipitation, samples containing equal amounts of protein were precleared with various irrelevant IgG or specific antibodies (2-5 mg/ml) overnight at 4°C with gentle rotation and subsequently incubated with Protein A/G agarose/sepharose beads at 4°C with gentle rotation. After 3 hr of incubation, agarose/sepharose beads were washed extensively with phosphate-buffered saline (PBS) 4 times, and proteins were eluted by boiling in 1× SDS sample buffer before SDS-PAGE electrophoresis.

### 2.9. Immunofluorescence

Following the appropriate treatment, cells in the plate were prepared as described previously [30]. Rabbit anti-ASC (10500-1-AP, Proteintech), anti-LC3 (sc-16755, Santa Cruz Biotechnology), anti-LC3, goat anti-NLRP3, and anti-pp65 antibodies were used for immunofluorescence. Donkey anti-mouse IgG, donkey anti-goat IgG, and goat anti-rabbit FITC conjugated antibodies were used as secondary antibodies. The nuclei were stained with DAPI at the concentration of 1 *μ*g/ml for 10 min. Confocal microscopy (LSM 880, Zeiss optics international trading co., LTD) was used for examination.

### 2.10. SiRNA Transfection

RAW264.7 cells were seeded in six-well tissue culture plates at a density of 2 × 10^5^ cells per well with 2 ml of antibiotic-free DMEM (10% FBS) medium. The cells were incubated at 37°C in a CO2 incubator until the cells were 60-80% confluent. This usually took 18-24 hr. The following solutions were prepared: Solution A: for each transfection, 2-8 *μ*l of siRNA duplex was diluted into 100 *μ*l of siRNA Transfection Medium. Solution B: for each transfection, 6 *μ*l of siRNA Transfection Reagent was diluted into 100 *μ*l of siRNA Transfection Medium. siRNA duplex solution (Solution A) was added directly to the dilute Transfection Reagent (Solution B) using a pipette. The solution was mixed gently by pipetting up and down, and the mixture was incubated for 15-45 min at room temperature. The cells were washed once with 2 ml of siRNA Transfection Medium. The medium was aspirated and immediately used in the next step. For each transfection, 0.8 ml of siRNA Transfection Medium was added to each tube containing the siRNA Transfection Reagent mixture (Solution A + Solution B). The solution was mixed gently, and the mixture was overlaid onto the washed cells. The cells were incubated for 5-7 hr at 37°C in a CO_2_ incubator. One milliliter of DMEM (20% FBS) was added without removing the transfection mixture. The cells were incubated for an additional 18-24 hr. The medium was aspirated and replaced with fresh DMEM (10% FBS) and cell cultivation continued for 24-72 hr. Then, the transfection efficiency was evaluated by Western blot to detect the expression of BECN1.

### 2.11. Statistical Analysis

Statistical analysis was performed by using GraphPad Prism 5 (GraphPad Software, La Jolla, CA, USA). *P* values were assessed using variance (ANOVA) one-way analysis, and results were given as the means ± standard deviations (SD). *p* < 0.05 was considered statistically significant.

## 3. Results

### 3.1. Effect of Guttiferone K on the Viability of RAW264.7 Cells

**Figure 1:**
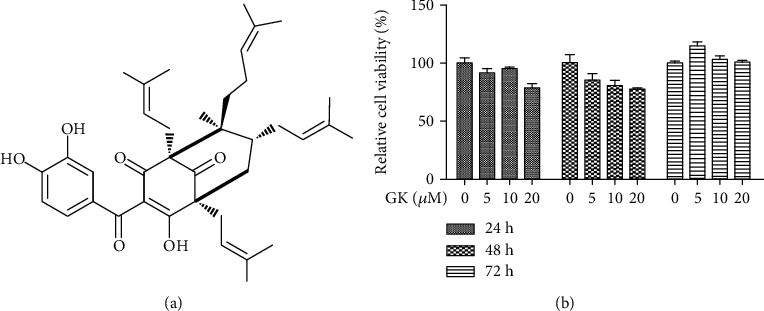
Effect of Guttiferone K on the viability of RAW264.7 cells. (a) The chemical structure of Guttiferone K. (b) A proliferation assay was conducted to assess the cytotoxic effect of Guttiferone K on RAW264.7 cells. RAW264.7 cells were treated with various concentrations of GK (0, 5, 10, 20 *μ*M) for 24, 48, and 72 hr. After the addition of CCK-8 reagent, the optical density of each well was determined at 450/650 nm. Data are shown as the means ± SD of three independent experiments.

To ensure that a safe concentration range of GK was used on the cells, the viability assay was conducted to evaluate potential drug-induced toxicity. The proliferation of RAW264.7 cells was tested using the CCK-8 kit. As shown in Figure 1, although GK (within 20 *μ*M) had a weak inhibitory effect on the viability of RAW264.7 cells during 24 hr, 48 hr, and 72 hr, the concentration of GK used in our subsequent experiments was 10 *μ*M, and the maximum treatment time on cells was 12 hours. Therefore, the concentrations of GK within 20 *μ*M were considered safe for cells during our observation and could be used for subsequent studies.

### 3.2. Guttiferone K Decreases the Production of Crucial Proinflammatory Mediators Induced by Mtb Infection

**Figure 2:**
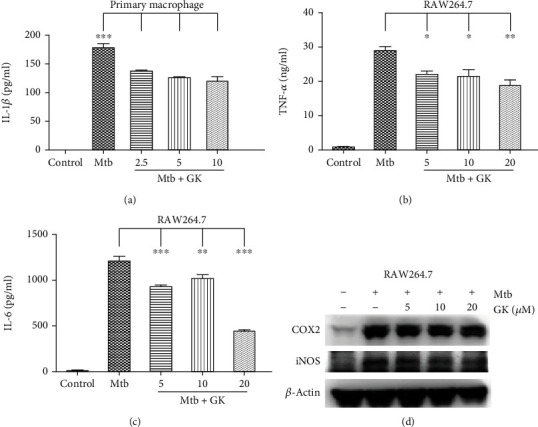
Guttiferone K inhibits Mtb-induced proinflammatory cytokines production. Primary peritoneal macrophage (a) cells or RAW264.7 (b–d) were infected with H37Ra and treated with different concentrations of GK (2.5, 5, 10, or 20 *μ*M) for 12 hr. The concentrations of IL-1*β* (a), TNF-*α* (b), and IL-6 (c) in the supernatants were measured by ELISA. (d) GK suppresses the expression of iNOS and COX2 induced by Mtb infection, and the protein levels of iNOS and COX2 were detected by Western blot. Data are shown as the means ± SD (*n*≧3). ^∗^*p* < 0.05, ^∗∗^*p* < 0.01, ^∗∗∗^*p* < 0.001 (one-way ANOVA).

Macrophages serve as the major host cell niche for Mtb. Additionally, as inflammatory mediator-producing cells, macrophages play an important role in inflammatory disease processes. We first observed the effect of GK on the production of vital inflammatory mediators in Mtb-infected macrophages. Different cytokines in the supernatants were measured by ELISA, and the expression of iNOS and COX2 was detected by Western blot. The results demonstrated that GK evidently inhibited Mtb-triggered IL-1*β*, TNF-*α*, and IL-6 secretion (Figures 2(a)–2(c)) in a concentration-dependent manner. Similarly, as shown in Figure 2(d), GK decreased the protein expression of iNOS and COX2, which are responsible for NO and PGE2 production, respectively. Thus, the results demonstrated that GK can downregulate the secretion of IL-1*β* (in Mtb-infected primary macrophages), which has been reported to be involved in TB immunopathology, as well as other critical cytokines including TNF-*α* and IL-6, and inhibits the production of iNOS and COX2 in Mtb-infected RAW264.7 cells.

### 3.3. Guttiferone K Inhibits NF-*κ*B Activation through the TLR/IRAK-1 Pathway

**Figure 3:**
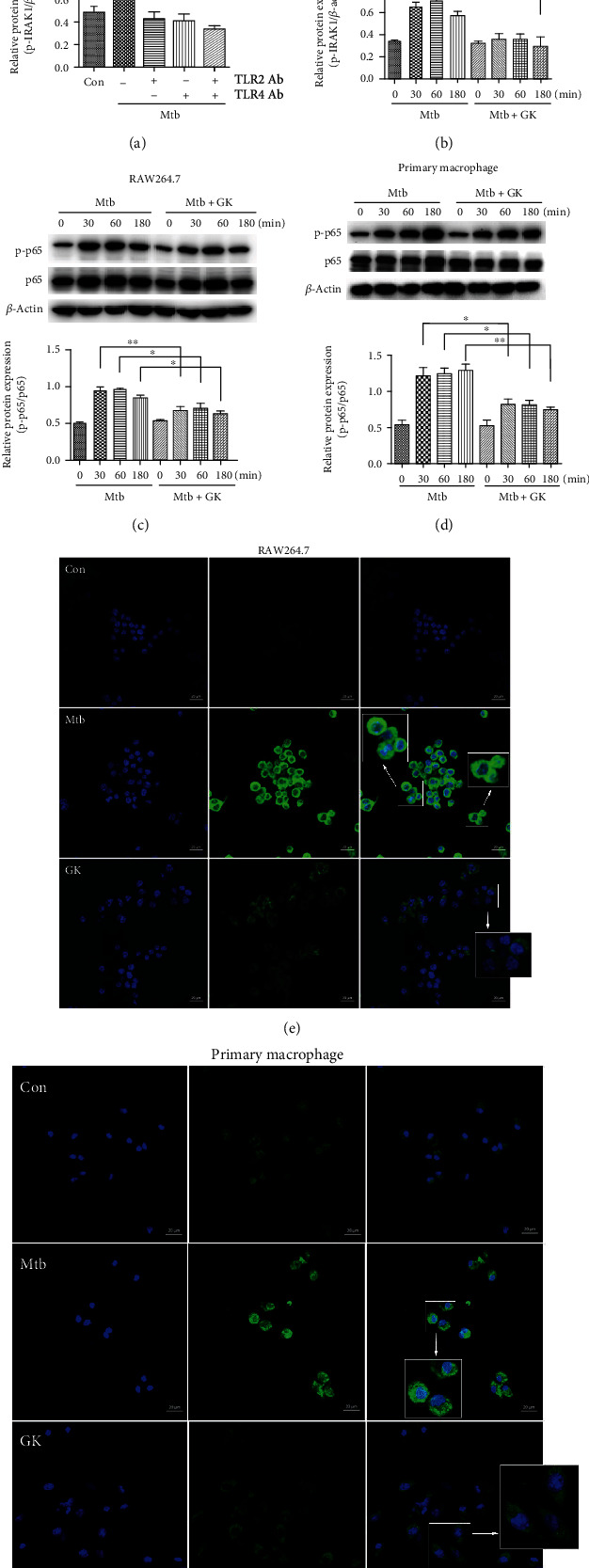
Guttiferone K inhibits Mtb-triggered activation of the NF-*κ*B signaling pathway by targeting the TLR/IRAK-1 pathway. (a, b) Western blot analysis of p-IRAK1 expression in RAW264.7 and primary peritoneal macrophage cells. (a) TLR2/4 antibodies were pretreated on RAW264.7 cells before Mtb infection; then, the production of p-IRAK1 at the protein level was detected by immunoblotting and normalized to *β*-actin. (b) Primary peritoneal macrophage cells were infected with Mtb for 0, 30, 60, and 180 min and correspondingly treated with GK (10 *μ*M) for 0, 30, 60, and 180 min; then, the production of p-IRAK1 at the protein level was detected by immunoblotting and normalized to *β*-actin. Data are representative of at least three independent experiments. (c, d) Evaluation of NF-*κ*B expression at the protein level. Western blot analysis of p-p65 expression in RAW264.7 cells (c) or in primary peritoneal macrophage cells (d) and normalized to total p65. (e, f) GK prevents the nuclear translocation of Mtb-induced NF-*κ*B. Confocal microscopy of RAW264.7 cells (e) or primary peritoneal macrophage cells (f) receiving different treatments immunostained with anti-pp65 (green) and DAPI (blue). Data are representative of at least three independent experiments, ^∗^*p* < 0.05, ^∗∗^*p* < 0.01 (one-way ANOVA).

TLRs are generally considered to be proinflammatory. The interaction of specific mycobacterial structures with TLRs will trigger the phosphorylation of downstream IRAK-1 instantly and subsequently recruit other important molecules in a signaling cascade leading to activation of the NF-*κ*B pathway, resulting in large amounts of inflammatory mediator expression and an uncontrollable inflammatory response. To observe whether TLR2/4 is related to the Mtb-induced inflammatory response, we first used a specific TLR2/4 antibody to block the TLR2/4 receptors of macrophages for 40 min before applying other treatments (except the control group and the Mtb group); then, the corresponding groups were infected with Mtb (MOI = 10) for 4 hr, then replaced fresh DMEM (10% FBS) to each group for another 12 hr. As shown in Figure 3(a) (RAW264.7 cells), when TLR2/4 receptors were blocked, the phosphorylation level of IRAK-1 significantly declined. Therefore, Figure 3(b) (primary peritoneal macrophages) shows that GK inhibited the IRAK-1 phosphorylation in a time-dependent manner. As expected, GK significantly inhibited the phosphorylation of p65 in both of RAW264.7 (Figure 3(c)) and primary peritoneal macrophages (Figure 3(d)) and prevented p-p65 transportation to the nucleus in both RAW264.7 (Figure 3(e)) and primary peritoneal macrophages (Figure 3(f)). Combined with the results shown in Figures 3(a) and 3(b), we concluded that Mtb induce the NF-*κ*B activation through TLR/IRAK-1, and GK showed an anti-inflammatory effect by targeting the TLR/IRAK-1 pathway.

### 3.4. Guttiferone K Shows No Significant Influence in the MAPK Inflammatory Pathway

**Figure 4:**
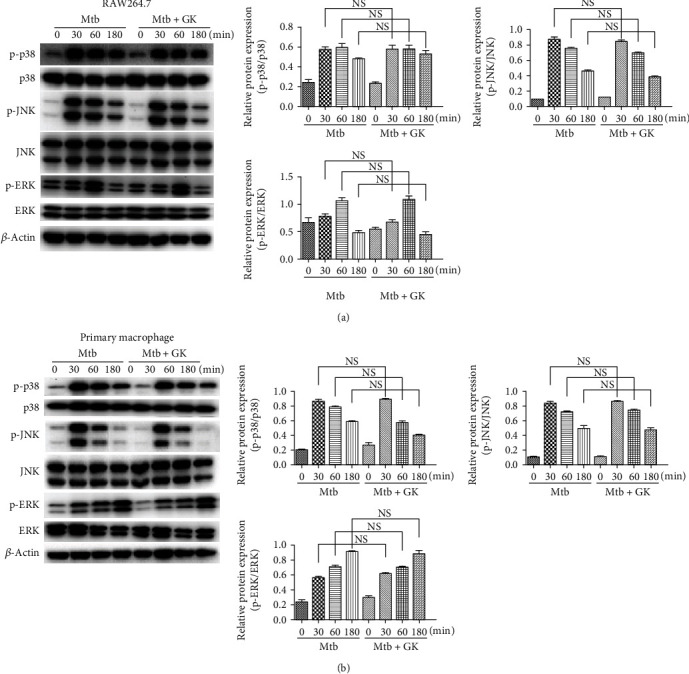
Guttiferone K has no effect on the MAPK pathway. RAW264.7 cells (a) or primary peritoneal macrophage cells (b) were infected with Mtb and treated with or without GK (10 *μ*M) for 30, 60, or 180 min. The protein levels of JNK, p-JNK, ERK, p-ERK, p38, p-p38, and *β*-actin were detected by Western blot and normalized to their respective nonphosphorylated total proteins. The results are representative of at least three independent experiments. NS: not significant.

MAPK and NF-*κ*B signalings are recognized as downstream of the TLR pathways and trigger proinflammatory responses in many inflammatory diseases, including TB. The results abovementioned suggested the inhibitory effect of GK on the NF-*κ*B pathway, and thus, the next study was designed to examine whether GK has a regulatory effect on MAPK signaling. As shown in Figure 4, Mtb infection induced the activation of the MAPK pathway, as evidenced by the marked elevated phosphorylation of p38, JNK, or ERK both in RAW264.7 (Figure 4(a)) and primary macrophages (Figure 4(b)). However, GK treatment had hardly any impact on the expression of Mtb-induced p-p38, p-JNK, or p-ERK on both of the cell lines. Thus, the current results suggested that GK exerts anti-inflammatory effects in Mtb-infected macrophages by targeting TLR/IRAK-1 signaling, then negatively regulating the downstream NF-*κ*B pathway without affecting the MAPK inflammatory pathway.

### 3.5. Guttiferone K Suppresses Mtb-Induced NLRP3 Inflammasome Activation

**Figure 5:**
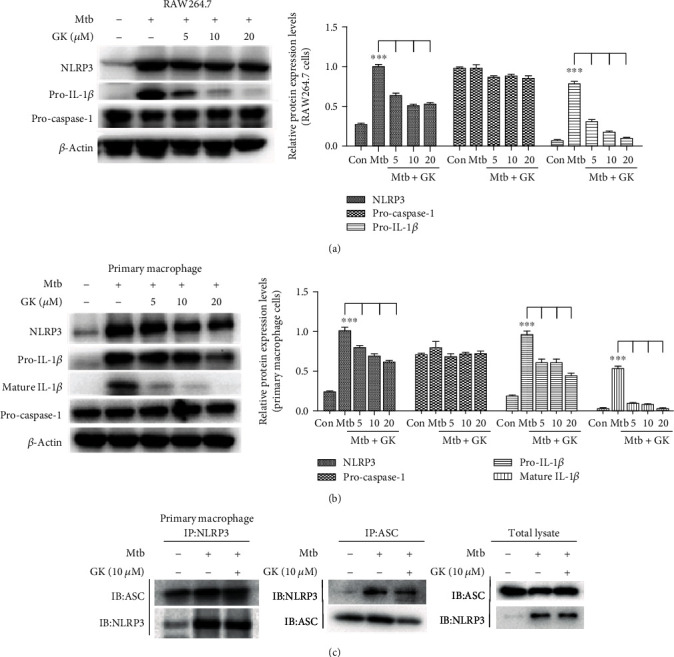
Guttiferone K significantly inhibits Mtb-induced NLRP3 inflammasome activation. (a, b) Levels of NLRP3, pro-IL-1*β*, and pro-caspase-1 expression in cell lysates and mature IL-1*β* in the supernatant were determined by Western blot in RAW264.7 (a) and primary peritoneal macrophage cells (b) and normalized to *β*-actin. (c) ASC or NLRP3 immunoprecipitates from primary peritoneal macrophage cells were immunoblotted for NLRP3 or ASC and reblotted for ASC or NLRP3, respectively. The results are representative of at least three independent experiments, ^∗∗∗^*p* < 0.001 (one-way ANOVA), compared with the Mtb group.

Inflammasome activation is an important posttranscriptional event to facilitate IL-1*β* release, and the NLRP3 inflammasome has been reported to contribute to inflammatory tissue damage during mycobacterial infection. We have observed the obvious inhibitory effect of GK on IL-1*β* (Figure 2(a)), so the next experiment was designed to detect the effect of GK on NLRP3 inflammasome. As expected, Western blot showed that Mtb induced the increased expression of NLRP3 and pro-IL-1*β* in both RAW264.7 cells (Figure 5(a)) and primary macrophages (Figure 5(b)). In contrast, GK treatment decreased NLRP3 and pro-IL-1*β* in a dose-dependent manner in both cell lines (Figures 5(a) and 5(b)) and inhibited the production of mature IL-1*β* (Figure 5(b)). The inhibitory effect of GK on NLRP3 and pro-IL-1*β* might be attributed to the GK-mediated suppression of TLR/NF-*κ*B, as NF-*κ*B signaling has been known to provide the first signal for NLRP3 inflammasome activation by promoting the expression of NLRP3 and pro-IL-1*β*. Moreover, Co-IP (primary peritoneal macrophages) showed that Mtb induced the association of NLRP3 with ASC, while GK interrupted this relation (Figure 5(c)); these data revealed that GK also inhibited the inflammasome assembly process. Taken together, this part of the results indicated that GK could suppress Mtb-induced NLRP3 inflammasome activation and subsequently triggered inflammation.

### 3.6. Guttiferone K Inhibits NLRP3 Inflammasome Activity by Inducing the Autophagy Process

**Figure 6:**
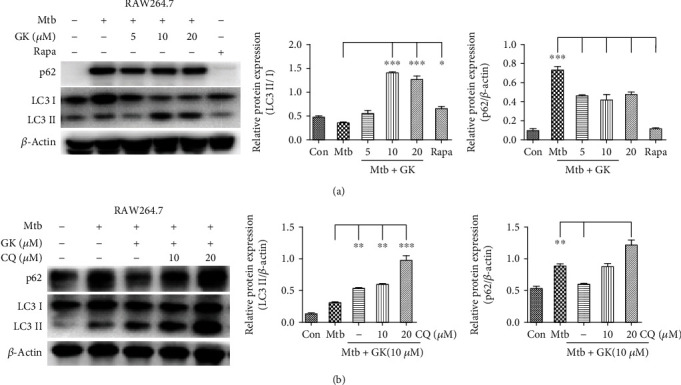
Guttiferone K can promote the activation of autophagy in Mtb-infected macrophages. (a) Western blot analysis of LC3 I/II and p62 expression in Mtb-infected RAW264.7 cells after treatment with different concentrations of GK (5, 10, 20 *μ*M) or rapamycin (1 *μ*g/ml) for 12 hr, normalized to LC3 I or *β*-actin. (b) Western blot analysis of LC3 I/II and p62 expression in Mtb-infected RAW264.7 cells after treatment with GK (10 *μ*M) or CQ (10, 20 *μ*M) for 12 hr, normalized to LC3 I or *β*-actin. The results are representative of at least three independent experiments, ^∗^*p* < 0.05, ^∗∗^p < 0.01, ^∗∗∗^*p* < 0.001 (one-way ANOVA), compared with the Mtb group.

**Figure 7:**
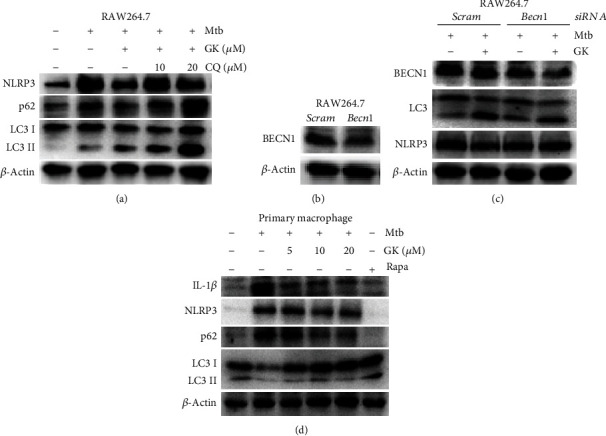
Guttiferone K restrains NLRP3 inflammasome activity depending on the autophagy process. (a) Western blot analysis of NLRP3, LC3 I/II, and p62 expression in Mtb-infected RAW 264.7 cells after treatment with GK (10 *μ*M) or CQ (10 or 20 *μ*M) for 12 hr. (b) Evaluation of si-BECN1 transfection efficiency by Western blot in RAW264.7 cells. (c) BECN1 knockdown downregulates LC3 and restricts the inhibitory effect of GK on NLRP3 in RAW264.7 cells. (d) Western blot analysis of IL-1*β*, NLRP3, LC3 I/II, and p62 expression in Mtb-infected primary peritoneal macrophage cells after treatment with different concentrations of GK (5, 10, 20 *μ*M) or rapamycin (1 *μ*g/ml). The results are representative of at least three independent experiments.

**Figure 8:**
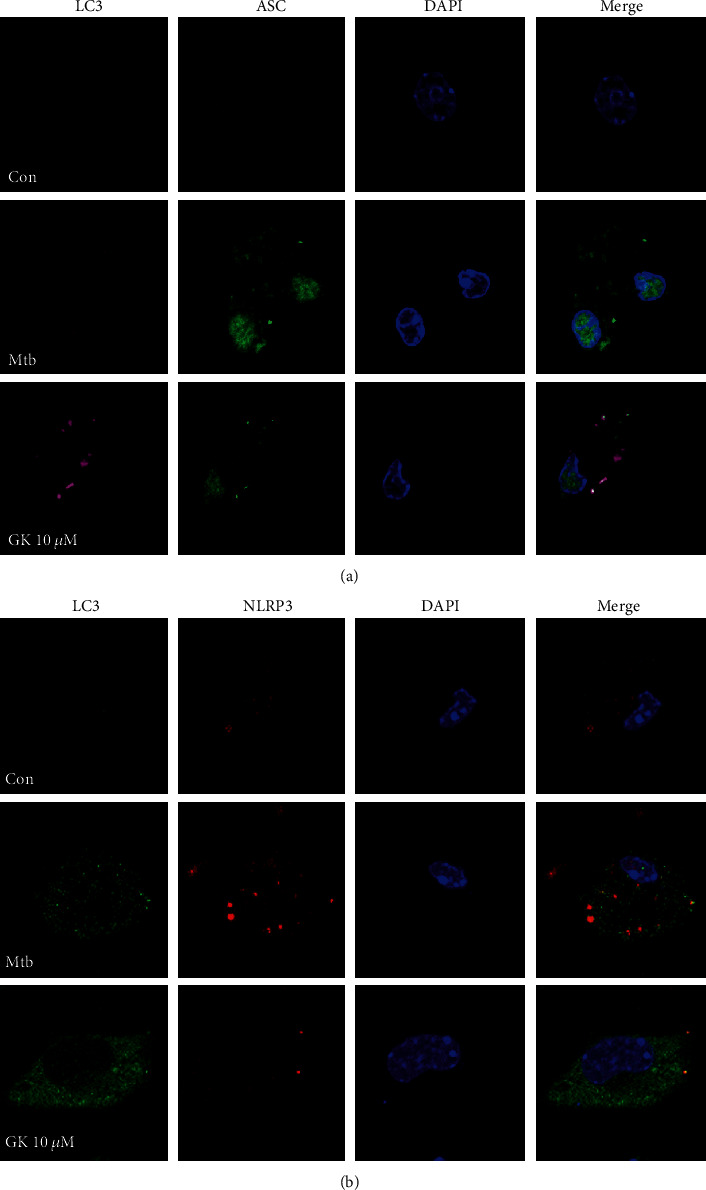
Guttiferone K promotes the colocalization of inflammasomes with autophagosomes. (a) Confocal microscopy of primary peritoneal macrophages with different treatments immunostained with anti-LC3 antibody (pink), anti-ASC antibody (green), and DAPI (blue). (b) Confocal microscopy of primary peritoneal macrophage cells with different treatments immunostained with anti-LC3 antibody (green), anti-NLRP3 antibody (red), and DAPI (blue). Experiments performed at least three times.

Autophagy has been shown to play an important role in regulating inflammasome activation through the removal of inflammasome-activating endogenous signals or the sequestration and degradation of inflammasome components. Previous studies showed that GK induced tumor cell autophagy and promoted tumor cell apoptosis [28, 31]. Therefore, we investigated the effect of GK on autophagy induction in Mtb-infected macrophages. As shown in Figure 6 (RAW264.7 cells), GK induced the activation of autophagy at different doses, and the concentration of 10 *μ*M triggered the most potent autophagy, evidenced by the detection of LC3I/II and p62 (biomarkers of autophagosome) (Figure 6(a)). Moreover, autophagic flux evaluation (Figure 6(b)) indicated that either 10 or 20 *μ*M chloroquine (CQ, an autophagy inhibitor) caused remarkable accumulation of LC3 I/II and p62 relative to individual GK treatment. These data proved that GK induced the activation of autophagy in Mtb-infected macrophages. To investigate whether GK-induced autophagy had inhibitory effect on NLRP3 inflammasome activity, we designed the following experiments. As shown in Figure 7(a) (RAW264.7 cells), when autophagy was disturbed by CQ treatment, GK could not induce an inhibitory effect on NLRP3 expression. Moreover, after knockdown of the Beclin 1 gene of RAW264.7 cells by a specific siRNA targeting *Beclin 1*, GK showed a decreased inhibitory effect on Mtb-induced NLRP3 expression (Figures 7(b) and 7(c)). In contrast, when autophagy was induced smoothly in primary peritoneal macrophages by GK treatment, GK exerted obvious inhibitory effects on NLRP3 expression or IL-1*β* production (Figure 7(d)). The results shown in Figure 7(d) that GK-induced autophagy in primary peritoneal macrophages further support the conclusion of Figure 6, which together with Figure 6 demonstrated that GK promotes the activation of autophagy both in RAW264.7 and primary peritoneal macrophages. To observe more intuitively the interaction between autophagy and inflammasome, we employed immunofluorescence using a confocal laser scanning microscope to observe the colocalization of LC3 with ASC or NLRP3. As shown in Figure 8 (primary peritoneal macrophages), Mtb induced the accumulation of ASC or NLRP3 protein, and GK treatment markedly decreased ASC specks and NLRP3 expression. Meanwhile, GK elevated the production of LC3 and induced the colocalization of LC3 with ASC or NLRP3. In summary, GK-induced autophagy activation inhibited the activity of NLRP3 inflammasomes through an autophagic degradative mechanism and restrained the secretion of IL-1*β*.

**Figure 9:**
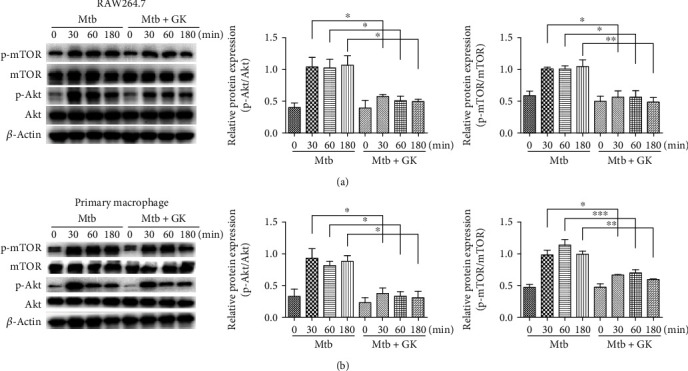
Guttiferone K inhibits the PI3K/Akt/mTOR pathway to play an anti-inflammatory role. (a) RAW264.7 cells or (b) primary peritoneal macrophage cells were infected with Mtb and treated with or without GK (10 *μ*M) for 30, 60, or 180 min. The protein levels of Akt, p-Akt (Ser 473), mTOR, and p-mTOR were detected by Western blot and normalized to nonphosphorylated total protein. The results are representative of at least three independent experiments, ^∗^*p* < 0.05, ^∗∗^*p* < 0.01, ^∗∗∗^*p* < 0.001 (one-way ANOVA).

These data revealed that GK exhibits positive anti-inflammatory effects on Mtb-infected macrophages by acting on the TLR/IRAK-1/NF-*κ*B pathway, inhibiting NLRP3 inflammasome and inducing autophagy activation to inhibit inflammasome activity. We next investigated the classical pathway on regulating autophagy, the Akt/mTOR signaling pathway, which is also the downstream of TLR signaling. Inhibition of Akt/mTOR is known to activate autophagy. As shown in Figure 9, GK treatment inhibited the phosphorylation of Akt (Ser473) and mTOR (Ser2448) in a time-dependent manner in both RAW264.7 cells (Figure 9(a)) and primary macrophages (Figure 9(b)). Thus, the results revealed that GK inhibited the TLR/Akt/mTOR signaling pathway to activate autophagy and has a role of anti-inflammation.

## 4. Discussion

Despite the availability of effective drugs against Mtb has been for more than 50 years, TB remains a major infectious disease worldwide. The deficiency of new drugs is a major obstacle to design new regimens against TB; HDT is emerging as a promising research field and is opening new avenues to develop new drugs for fighting TB. Research has shown that controlling the production of mature IL-1*β* significantly alleviated Mtb-induced host lung and spleen inflammatory damage [17] and applying anti-inflammatory drugs for other diseases as adjuvant TB treatments have achieved considerable results [18]. The present study first observed the effect of Guttiferone K on the regulation of Mtb-induced inflammation disorder. Guttiferone K is a bioactive polycyclic polyprenylated acylphloroglucinol (PPAP) found at high concentrations in the edible fruits of *Garcinia yunnanensis*. Species of *Garcinia* (Guttiferae) have been used to combat malaria in traditional African and Asian medicines [32, 33]. Studies have shown that Guttiferone K exerts favorable anticancer roles in different tumor cells. Based on the existing research, there is no study reported thus far on whether Guttiferone K is involved in playing regulatory roles during TB. This work was designed based on the control of Mtb-induced dysregulated inflammation to find bioactive molecules and provide a possible alternative for the development of new HDT drugs against TB.

There some studies unanimously suggested that Mtb infection can induce the activation of NLRP3 inflammasome, leading to mature IL-1*β* production in infected macrophages [34, 35]. Consistently, our work demonstrated that Mtb induced the NLRP3 inflammasome activation in the infected macrophages as the Figure 5 showed. ESAT-6 has been shown to be an essential element for activating the NLRP3 inflammasome. H37Ra strain has a defect in ESAT-6 secretion due to the mutation in the phoP gene, although the intracellular ESAT-6 concentration is similar in both H37Rv and H37Ra strains. We speculate that the potential mechanism for activating H37Ra-induced NLRP3 inflammasome may be that there are some lysed strains in the in vitro cultures release ESAT-6, thereby activating the inflammasome. IL-1 is a central proinflammatory cytokine that initiates and amplifies the inflammatory response process through inflammatory cascades by stimulating various immunological and inflammatory cells to synthesize proinflammatory cytokines, including TNF-*α* and IL-6 [36], or attracting chemokines and neutrophil chemotaxis to the infected site [37]. However, this defense response results in severe damage to host tissue while fighting against Mtb [38]. In the current study, our results demonstrated that Guttiferone K significantly inhibited the production of proinflammatory mediators that have been proved to participate in the process of pathological damage caused by uncontrolled inflammation during TB [39–41] and restraining the activation of NLRP3 inflammasome. Thus, Guttiferone K possesses a favorable inhibitory effect on regulating Mtb-triggered uncontrolled inflammation.

In our previous research, we have proved that Guttiferone K can activate autophagy in several cancer cell lines and accelerate cancer cell death under nutrient starvation in an autophagy-dependent manner [28]. Current reports have consistently proven that autophagy can negatively regulate the process of inflammation by inhibiting the release of mature IL-1*β* [19, 20] and protect the host from Mtb-induced excessive inflammation [16, 21]. Autophagy can inhibit inflammasome activity by clearing the endogenous stimuli that induce inflammasome activation [42, 43] or directly degrading the components of the inflammasome [44]. Consistent with previous results, our present work demonstrated that Guttiferone K also functions to promote autophagy in Mtb-infected macrophages and inhibits the activation of NLRP3 inflammasome as well as the production of mature IL-1*β* depending on autophagy. Additionally, Guttiferone K showed killing effect on intracellular invading germs by inducing autophagy activity (data not shown). Thus, Guttiferone K has a prominent advantage to be a promising candidate for the development of adjunctive HDT anti-TB drugs.

**Figure 10:**
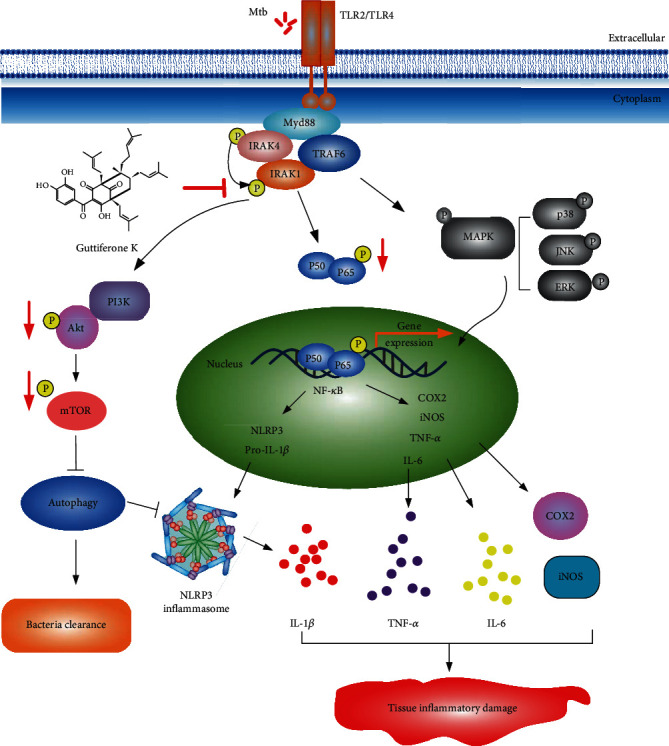
Molecular mechanism by which Guttiferone K exerts anti-inflammatory effects in Mtb-infected macrophages. Through TLR2/TLR4 recognition, Mtb intrudes into the macrophage and then accelerates IRAK-1 phosphorylation to activate its downstream signaling pathways, including NF-*κ*B, MAPK, and Akt/mTOR, causing inflammasome activation, increased production of inflammatory mediators, and autophagy inhibition, ultimately resulting in tissue damage. Guttiferone K targets the inhibition of IRAK-1 phosphorylation and then drives the inhibition of downstream NF-*κ*B and Akt/mTOR, reducing the expression of inflammatory factors and promoting cell autophagy to alleviate inflammation-mediated tissue damage induced by Mtb.

Guttiferone K has been shown to inhibit Mtb-induced dysfunctional inflammation; the possible molecular regulatory mechanisms involved in the effective function of Guttiferone K need to be further explored. Considering that TLRs (especially TLR2/4) play critical roles in the recognition of Mtb and regulation of the Mtb-induced inflammatory response, we designed next experiments to investigate the effect of Guttiferone K on TLR/IRAK-1. Results demonstrated that Mtb can activate the phosphorylation of IRAK-1 through TLR2/4, and Guttiferone K suppressed the IRAK-1 phosphorylation, which relies on TLR2/4. After TLR2/4 was blocked with specific antibodies, p-IRAK-1 suppression by Guttiferone K was weakened. Thus, we conclude that Guttiferone K may act on the TLR/IRAK-1 pathway playing anti-inflammatory effects. Next, we inspected the effect of Guttiferone K on two important inflammatory signaling pathways downstream of TLR signaling: the NF-*κ*B and MAPK pathways. The NF-*κ*B signaling pathway is well known to play important roles in promoting the inflammatory response and contributing to NLRP3 inflammasome activation. Results revealed that Guttiferone K exhibited an outstanding inhibitory effect on this pathway by restraining the phosphorylation of NF-*κ*B and its nuclear translocation. However, Guttiferone K rarely affects the phosphorylated JNK, ERK, or p38. The obtained results indicated that Guttiferone K played an anti-inflammatory effect by targeting TLR/NF-*κ*B but not TLR/MAPK. The Akt/mTOR signaling is the recognized pathway that negatively regulates autophagy and is also downstream of the TLR signaling pathway. Our previous study on different types of cancer cells which suffering nutrient stress had proved that the molecular mechanism of Guttiferone K-induced autophagy involved Akt/mTOR inhibition. Consistent results were obtained, and our current data indicated that Guttiferone K obviously inhibits the phosphorylation of Akt (Ser473) and mTOR (Ser2448). These data were a slightly different from our previous results in nutrient stress-treated tumor cells, in which Guttiferone K worked through multiple signaling pathways to activate autophagy, including the upregulation of reactive oxygen species (ROS) and JNK phosphorylation, except for Akt/mTOR inhibition [29]. The causes for this discrepancy may due to cell model differences and nutrient environment. Thus, the results indicated that Guttiferone K plays roles in restraining inflammation by targeting TLR/IRAK-1 signaling and then inhibiting the NF-*κ*B and Akt/mTOR pathways to relieve Mtb-triggered inflammatory tissue damage (Figure 10).

In conclusion, by inhibiting IRAK-1 phosphorylation and then driving the inhibitory effects on downstream NF-*κ*B and Akt/mTOR signaling, Guttiferone K showed a significantly suppressive effect on the expression of inflammatory factors and promoted cell autophagy to alleviate inflammation-mediated tissue damage induced by Mtb infection (Figure 10). Thus, our data demonstrated that Guttiferone K has a remarkable anti-inflammatory effect on Mtb-infected macrophages, and this endows Guttiferone K with the potential value as a candidate in the design of new adjuvant anti-TB HDT drugs. There may be some controversy about the use of H37Ra strain (an attenuated Mtb strain) to establish the Mtb infection model in this study. H37Rv and H37Ra are two widely used Mtb laboratory strains, and both were derived from the Mtb H37 parent strain. There are unique advantages of using the attenuated Mtb H37Ra strain. Primarily, biosafety level II is sufficient when using the attenuated strain for experiments, which makes the experiments more cost-efficient. Although some consider H37Rv the preferred strain for TB experimental research, a recent study [45] showed that Mtb H37Ra and H37Rv strains have equivalent minimum inhibitory concentrations (MIC) to most antituberculosis drugs. In our future research, we will focus on the cotreatment of Guttiferone K with first-line anti-TB drugs as well as studies in animal models. Drugs or small bioactive molecules, such as Guttiferone K, that possess favorable anti-inflammatory effects and manipulate host cellular defense mechanisms, such as autophagy, may provide new opportunities to combat infection by intracellular pathogens like Mtb.

## Data Availability

The data used to support the findings of this study are available from the corresponding author upon request.
